# Suturing Divided Nerve Trunks

**Published:** 1880-08

**Authors:** Lewis S. Pilcher


### SUTURING DIVIDED NERVE TRUNKS.

BY LEWIS S. PILCHER, M. D.

Dr. L. S. Pilcher called the attention of the Brooklyn Medical Society to the possibilities in the treatment of divided nerve trunks, which were to be realized by bringing together the separated ends and uniting them by suture. He claimed that in all cases of division of nerve trunks there was no reason to expect any evil effect from such practice. It was true that many instances of spontaneous repair and reunion of divided nerves had been recorded, but in no one case could a surgeon expect such a result; on the contrary, the rule was that degenerative changes rapidly ensued at the divided points, diminishing the probabilities of restoration of the function of the nerve more and more as time elapsed. The best results might be expected in cases of wounds in which nerve trunks had been divided, by at once uniting the separated ends by sutures. In many cases the sutures need be inserted only in the nerve sheath, but if necessary to obtain sufficient hold, they might be made to traverse the entire thickness of the nerve. In support of his opinion he cited first a case reported by Mr. J. W. Hulke to the Clinical Society of London, Feb. 13, 1880, and recorded in the *British Medical Journal* of Feb. 21, 1880, which was as follows: A man received a deep gash across the front of the forearm just above the wrist, by which the median nerve, radial artery, and several tendons were severed. Five weeks later, July 23, 1879, there having been no return of nerve function, the nerve was exposed by dissection. The ends were found three-quarters of an inch apart, the upper bulbous, the lower pointed. The two ends were refreshed, the upper end pulled down, and the ends fastened together by four stitches of very fine silk passed through the sheath; the forearm and hand were then fixed upon a splint. Three weeks later he was discharged, having regained sensibility in the parts before numb, except in the last phalanges of the index and middle fingers. Later, further improvement took place.

Second, a similar case which Mr. Howard Marsh, in discussing the preceding case, stated had occurred at St. Bartholomew's Hospital, in which, though the nerve had been injured for some time, the function began to return within twenty-four hours after union.

Third, a most remarkable case of suture of the great sciatic nerve, by Prof. von Langenbeck, of Berlin, related by Dr. Gaston Dupré in a letter to the *Journal de Medecine* of Brussels, 1877.

A man fell upon a cleaver and sustained a deep cut in the back of the right thigh, at a point a little above the lower third; loss of motion and sensation in the limb resulted. The wound suppurated, and finally healed at the end of two months. The patient walked with great difficulty, his leg wasted more and more; there was persistent insensibility. Massage, douches and electricity were applied without success. At the end of two years he presented himself at the clinic of Prof. von Langenbeck. The right leg was much wasted; examination by means of needles plunged deeply into the flesh showed the existence of a zone of complete insensibility occupying the outer half of the foot and leg as high up as the upper third of the leg. In walking, the patient halted badly, dragging his flaccid leg and resting on the internal border of the foot, upon the external border of which an ulcer was developing. Upon the back of the thigh, about its lower third, was discernible a large transverse cicatrix. The professor made a long incision perpendicular to the cicatrix, and exposed much cicatricial connective tissue; dividing this carefully, he came upon the peripheral end of the nerve at the lower angle of the incision; this end was slightly swollen. Pursuing his search at the upper angle of the incision, he found, with much difficulty, buried in the midst of a large mass of cicatricial tissue, a large swelling of the size of a good-sized thumb ; it was the central end of the nerve. The two ends were distant from each other five centimetres (2 in.) The professor expressed surprise at such a great separation of the two ends of the divided nerve. He drew attention to how much greater was the swelling of the central than of the peripheral end of the nerve. It would be necessary, first of all, to determine that the peripheric end had not become entirely transformed into a fibrous and granulo-fatty cord. For this end, he cut away a certain length of the slender swelling, and ended by finding nerve fibres intact; the same course was pursued with the central end, so that the nerve tissue of the two parts could be placed in immediate contact; then exposing the peripheral end for some distance, and flexing the knee strongly, he brought the two ends of the nerve together. This done, the ends were fastened together by two catgut sutures, which were cut off near the knots ; some points of suture were placed in the skin, and finally a bent guttapercha splint applied to the anterior face of the limb, so as to maintain the flexion of the knee. No unpleasant symptoms followed this operation. During the next and the following days the patient experienced but little pain ; a very slight elevation of temperature, the immediate result of the operation, soon disappeared. At the end of two months, when the case was reported, sensation had been restored to such an extent over the whole part before insensible, that the patient could indicate precisely a point where a slight prick of the skin was made. Motion, however, was yet in abeyance.

The uniform benefits and freedom from dangerous or unpleasant complications attending these cases, encourage a more general practice of immediately fastening together by sutures the ends of divided nerve trunks. An extensive test of this method of procedure in the proper cases is urged.—*Annals of Anatomical and Surgical Society, Brooklyn.*

